# Common genomic features of *Campylobacter jejuni *subsp. *doylei *strains distinguish them from *C. jejuni *subsp. *jejuni*

**DOI:** 10.1186/1471-2180-7-50

**Published:** 2007-05-29

**Authors:** Craig T Parker, William G Miller, Sharon T Horn, Albert J Lastovica

**Affiliations:** 1 Produce Safety and Microbiology Research Unit, Agricultural Research Service, US. Department of Agriculture, Albany, CA 94710 USA; 2 Department of Biotechnology, University of the Western Cape, Bellville 7535, South Africa

## Abstract

**Background:**

*Campylobacter jejuni *has been divided into two subspecies: *C. jejuni *subsp. *jejuni *(*Cjj*) and *C. jejuni *subsp. *doylei *(*Cjd*). Nearly all of the *C. jejuni *strains isolated are *Cjj*; nevertheless, although *Cjd *strains are isolated infrequently, they differ from *Cjj *in two key aspects: they are obtained primarily from human clinical samples and are associated often with bacteremia, in addition to gastroenteritis. In this study, we utilized multilocus sequence typing (MLST) and a DNA microarray-based comparative genomic indexing (CGI) approach to examine the genomic diversity and gene content of *Cjd *strains.

**Results:**

A geographically diverse collection of eight *Cjd *strains was examined by MLST and determined to be phylogenetically distinct from *Cjj *strains. Microarray-based CGI approach also supported this. We were able to demonstrate that *Cjd *strains exhibited divergence from *Cjj *strains NCTC 11168 and RM1221 in many of the intraspecies hypervariable regions. Moreover, multiple metabolic, transport and virulence functions (e.g. cytolethal distending toxin) were shown to be absent in the *Cjd *strains examined.

**Conclusion:**

Our data demonstrate that *Cjd *are phylogenetically distinct from *Cjj *strains. Using the CGI approach, we identified subsets of absent genes from amongst the *C. jejuni *genes that provide clues as to the potential evolutionary origin and unusual pathogenicity of *Cjd*.

## Background

*Campylobacter jejuni *consists of two subspecies: *C. jejuni *subsp. *jejuni *(*Cjj*) and *C. jejuni *subsp. *doylei *(*Cjd*). *Cjd *strains originally were identified as gastric campylobacters from human gastric biopsies [1] and nitrate-negative campylobacters from pediatric patients with gastroenteritis [2]. The major phenotypic characteristic commonly used to distinguish *Cjd *strains from *Cjj *strains, is the inability of *Cjd *to reduce nitrate. Other phenotypic characteristics also associated with *Cjd *include variable growth at 42°C [3], high susceptibility to cephalothin [3], and the absence of γ-glutamyl transferase (GGT) and L-arginine arylamidase activity [4]. However, like *Cjj*, all *Cjd *strains are hippuricase positive.

In clinically-related aspects,* Cjd *strains also differ from *Cjj*. First, *Cjd *strains cause both gastritis [1,5] and enteritis [3,6-8], and often are isolated from pediatric patients [3,7-9]. Second, *Cjd *strains are isolated more often from blood cultures than from stool cultures [3]. Moreover, Morey reported that *Cjd *was isolated from 85.2% of *Campylobacter*/*Helicobacter*-related bacteremia cases in Australia during a five-year period [9]. Additionally, *Cjd *was isolated from 9.7% of the *Campylobacter*-positive stool cultures but 24% of the *Campylobacter*-positive blood cultures [10], obtained at Red Cross Children's Hospital, Cape Town, during the years 1977–1995.

Despite the unusual clinical symptomatology, *Cjd *is isolated infrequently and few strains exist (compared to *Cjj*) for this subspecies. One possible reason is that many clinical laboratories do not characterize *Campylobacter *isolates past the genus level or subspeciate *C. jejuni *isolates as *Cjj *or *Cjd*. Moreover, normal *Cjj *culturing methods select against *Cjd *that are susceptible to cephalothin and exhibit variable growth at 42°C. This appears to be supported by the fact that in South Africa, *Cjd *strains represent a significant proportion of the total campylobacters isolated from human clinical samples; where 16% of the non-*Cjj*/*coli Campylobacter *isolates were *Cjd*. These isolates were obtained using the Cape Town Protocol [3,11] which uses passive filtration through a 0.65 μM membrane filter, growth at 37°C and no antibiotic selection.

Multilocus sequence typing (MLST) [12-17] and microarray-based comparative genomic hybridization (CGH) [18-23] have been utilized by several groups to assess genomic diversity and index the gene content of hundreds of *C. jejuni *strains. In particular, these CGH or comparative gene indexing (CGI) studies identified regions of intraspecies hypervariability, such as the lipooligosaccharide biosynthesis (LOS), capsular biosynthesis (CAP), flagellar modification (FM), and DNA restriction/modification (R/M) loci [18-23]. Furthermore, DNA sequencing of certain intraspecies hypervariable loci identified diversity in both the DNA sequence of common genes and the gene composition at each locus. [24-27].

In this study, we have exploited the sequencing of housekeeping genes (MLST) and DNA microarray-based CGI to examine the genomic relationship between *Cjd *strains, to determine the relationship between *Cjd *and *Cjj *strains, and to identify possible genomic factors that may have contributed to the emergence of *Cjd *strains. The MLST indicated that *Cjd *strains in our geographically diverse collection are highly related. Moreover, CGI analysis identified genomic regions that are commonly absent from *Cjd *strains and may contribute to their pathogenic characteristics.

## Results and discussion

### Sequence typing of *Cjd *strains

**Figure 1:**
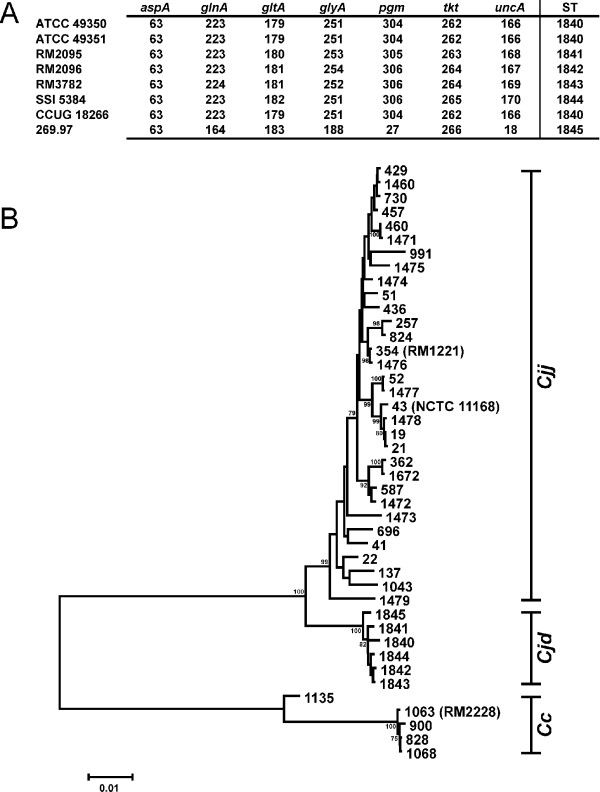
*Cjd *allelic profiles and phylogenetic relationships among *C. jejuni *subspecies and *C. coli *based on MLST.A. *Cjd *allelic profiles and MLST STs. B. Phylogenetic relationship between the six *Cjd *STs and representative *Cjj *and *C*. *coli *(*Cc*) STs. Allele sequences for each ST were first concatenated and then aligned using CLUSTALX. The dendogram was constructed using the neighbor-joining algorithm and the Kimura 2-parameter distance estimation method. Bootstrap values greater than 75%, generated from 500 replicates, are shown at the nodes. The scale bars represent substitutions per site. Numeric labels represent STs; the STs of the sequenced *Cjj *and *Cc *strains are indicated by the strain name in parentheses. Phylogenetic analyses were performed using MEGA version 3.1 [47]. The *C. jejuni *strains in panel B were identified to the subspecies level using a novel multiplex PCR method [35].

Multilocus sequence typing (MLST) of the eight *Cjd *strains listed in Table 1 identified six novel sequence types (STs) (Fig. 1A). Prior to this study, no *Cjd *STs were present (or at least STs from strains identified definitively as *Cjd*) in the *Campylobacter jejuni/coli *MLST database. It was intriguing that all eight *Cjd *strains contained the same *aspA *allele, *aspA63*, considering the geographical and temporal range of the strains. However, *aspA *has been shown previously to be associated also with a subgroup of campylobacters (e.g. the "jejuni-like" *aspA103 *allele in cluster II *C. coli *strains from turkeys [28]); therefore, it is possible that *aspA *is linked to a locus important for colonization or virulence, and that conservation at this locus is maintained by interstrain recombination. The *aspA63 *allele has been identified only three times before, in STs ST-6, ST-1458 and ST-2532. The phylogenetic relationship between the six *Cjd *STs and representative *Cjj *and *C. coli *(*Cc*) STs was also determined (Fig. 1B). The housekeeping gene sequences of each ST were concatenated and aligned. The *Cjd *strains form a distinct clade within the resulting dendogram supporting their subspecies designation. A similar organization of *Cjd *strains into a distinct cluster was observed previously using AFLP [29,30] and were observed in this study also by comparative genomic indexing (see below).

The presence of a single *Cjd *cluster invites the possibility that *Cjd *strains form a discrete group within the species and that no (or at best minimal) genetic exchange occurs between the two subspecies. Unfortunately, the resolution of such hypotheses by this study are hindered by the small size of the MLST-typed *Cjd *sample set (N = 8) and the high frequency of novel MLST alleles among the *Cjd *strains. With the exception of strain 269.97, the *Cjd *strains characterized in this study each possess six novel MLST alleles, with the seventh allele being the *aspA63 *allele described above. Thus, eBURST analysis, using 2532 *C. jejuni *(*Cjj *and *Cjd*) STs and a group definition of four common alleles, was not particularly informative (data not shown); ST-1842 and ST-1843 formed a two-ST clonal complex, ST-1845 (represented by strain 269.97) formed a clonal complex with ST-1458, and the remaining three *Cjd *STs were singletons. Although the presence of ST-1458 in the same clonal complex as 269.97 might indicate lateral gene exchange between the two subspecies, it is unclear from the data available if the strain representing ST-1458 is a *Cjj *or a *Cjd*. The same could be said, presumably, for a large number of strains within the *C. jejuni *MLST database that may not have been identified to the subspecies level. Therefore, to determine the nature of the origin and/or evolution of the *Cjd *subspecies and to determine whether *Cjd *alleles arise through the accumulation of point mutations or through a more rapid mechanism of exchange and recombination, additional experiments and analyses will be necessary. Such experiments would entail the characterization of a much larger and geographically diverse set of *Cjd *strains and the further identification of strains within the *C. jejuni *MLST database to the subspecies level. Characterization of additional *Cjd *strains might explain also the noteworthy conservation of the *aspA63 *allele within the subspecies.

### Comparative genomic indexing of the *Cjd *strains

We further examined the genomic diversity of these eight *Cjd *strains more comprehensively by comparative genomic indexing (CGI) analysis. The CGI analysis allowed the assessment of gene content for each *Cjd *strain relative to the multi-strain *C. jejuni *DNA microarray, described previously [23], that comprises 1530 genes from NCTC 11168 and 227 genes from RM1221. Genomic DNAs from both NCTC 11168 and RM1221 were used as a reference DNA mixture and competitively hybridized with genomic DNA from each of the *Cjd *strains. We observed that 23.4% (418 of 1786) of the genes represented on the microarray were highly divergent or absent (trinary score of 0) in at least one *Cjd *strain and 262 of these genes were absent from all of 8 of the *Cjd *strains in this study. In comparison, we observed 21.5% (385 of 1786) of the genes were highly divergent or absent in at least 1 of 35 geographically and temporally diverse *Cjj *strains from both humans and animals using this microarray [23]. The CGI data sets as trinary scores for the *Cjd *strains are available as an additional table [see Additional file 1].

**Figure 2:**
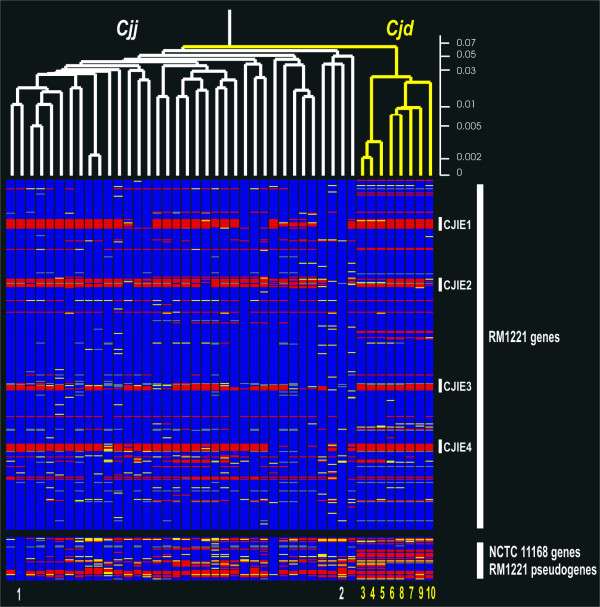
Comparison of *Cjd *and *Cjj *strains by cluster analysis of CGI results. The numbers at the bottom of the figure correspond to the *Cjd *strains in Table 1. Details of the diverse collection of *Cjj *strains have been described previously [23]. The genes are represented in the order of their positions from the RM1221 and are followed by the NCTC 11168 genes that include RM1221 pseudogenes. The four CJIEs from RM1221 and the capsular polysaccharide biosynthesis regions (HV 13) from RM1221 and NCTC 11168 are indicated. The gene status is color-coded: blue, present; yellow, divergent/unknown; red, absent and gray, no data. For cutoffs of absence and presence predictions, refer to *Materials and Methods*. An average linkage hierarchical clustering of the *C. jejuni *strains was compiled in GeneSpring version 7.3 from the CGI data for each element with the standard correlation and bootstrapping. *Cjd *strains are labeled in yellow and *Cjj *strains are labeled in white. The scale for distance score is also shown.

Using cluster analysis, we examined the genomic relationship between the *Cjd *strains and these 35 diverse *Cjj *strains that were analyzed previously [23]. Figure 2 depicts the relationship among the strains using a standard correlation function and bootstrapping (see Material and Methods) where the linkage distance between strains is represented by branch lengths in the resulting hierarchical cluster. These microarray-based CGI results at the whole genome level demonstrate that the *Cjd *strains formed a discrete cluster, correlating well with our MLST dendogram.

### Intraspecies hypervariability regions and genomic elements

As with *Cjj *strains, the majority of genes that were divergent in the *Cjd *strains were contained within described regions of intraspecies plasticity (PR)/hypervariability (HV) [e.g. HV regions 1–18 [22,23] and regions PR1-PR7 [21]. The *Cjd *strains exhibited identical presence/absence patterns for genes within 11 of the 18 intraspecies hypervariability regions described recently [23]. Interestingly, the genes Cj0728-Cj0734 within HV region 9 that were shown recently to be phosphate-regulated [31] were absent or highly divergent in all of the *Cjd *strains examined. It should be noted that the *Cjd *strains were not all identical at the hypervariable regions related to surface structures, including the lipooligosaccharide (LOS) biosynthesis locus (HV region 11) [21-24,32], the flagellar modification locus (HV region 12) [21-23,33], and the capsular polysaccharide biosynthesis locus (HV region 13) [21-23,25]. Furthermore, all of the *Cjd *strains were distinct from both NCTC 11168 and RM1221 in these three HV regions. This suggests that the *Cjd *strains do not produce one distinct set of surface molecules, nor do they produce the same surface molecules observed for either NCTC 11168 or RM1221.

We previously demonstrated the occurrence of four *Campylobacter jejuni *integrated elements (CJIE), similar to those identified in RM1221, in other strains of *C. jejuni *[23]. The CJIE1, CJIE2 and CJIE4 show similarity to bacteriophage while CJIE3 may be an integrated plasmid. For *Cjd *strains, fewer than 3 genes from the *Campylobacter *Mu-like phage (CJIE1) or CJIE4 were present. We observed that strain RM4099 possessed a majority of the genes within CJIE2 described in strain RM1221 [23,34], while *Cjd *strains RM1512, RM1513 and RM4098 possessed a smaller number of genes within CJIE2 (Fig. 2). Although not visible in Figure 2, these same strains (RM4099, RM1512, RM1513 and RM4098) also possessed a cluster of genes from CJIE3 (Cje1093-Cje1100), suggesting the possibility that a plasmid (integrated or otherwise) is present in these strains.

### Common deletions in *Cjd *strains

The *Cjd *strains also had missing genes outside the *C. jejuni *intraspecies hypervariable regions. Indeed, 46 genes not identified previously within the *C. jejuni *hypervariable regions, but missing from at least half of the *Cjd *strains, is presented in Table 2. Of these, 21 genes were absent from all *Cjd *strains (e.g. Cj0005c and Cj0091) and 8 genes were detected in only one *Cjd *strain (e.g.Cj0555 and Cj0636). The largest clusters of highly divergent loci contained three genes (Cj0201c-Cj0203 and Cj1040c-Cj1042c), with the remainder distributed uniformly throughout the chromosome, assuming genomic positions similar to the two sequenced *Cjj *strains. The majority (32/46; 70%) of the *Cjd *highly-divergent loci encode proteins with unknown or general function. The remaining genes, such as *argBC*, *napAB*,*proA*, *dcuB*, *ceuCD*, and *sdaC*, mostly encode metabolic or transport proteins. Furthermore, the microarray results suggest that the observed absence of nitrate reductase activity in *Cjd *strains is due most likely to deletions in *napA *and/or *napB*. We have confirmed recently these *nap *mutations by PCR and sequencing and our evidence strongly suggests that *Cjd *arose from a single evolutionary event, i.e. the *napA *deletion, with the divergence at *napB *occurring subsequently [35]. Despite these results, these metabolic and transport-related genes provide too few clues into their possible role in *Cjd *pathogenicity.

The total absence of the virulence gene *cdtA *(and near-total absence of *cdtB*) among the *Cjd *strains, however, may be related to pathogenicity (Table 2, Cj0078c and Cj0079c). The *cdt *genes encode subunits of the cytolethal distending toxin (CDT), indicating that *Cjd *strains do not produce this toxin. As CDT has been associated with virulence properties in *C. jejuni *[36-39], and *Cjd *has been isolated almost exclusively from human clinical samples, it is intriguing to speculate what role loss of a virulence factor plays in *Cjd *pathogenicity. Specifically, CDT from *C.jejuni *has been shown to arrest eukaryotic cells in the G_2_/M phase of the cell cycle [36,38] and induces the release of the proinflammatory cytokine, interleukin-8 (IL-8) [37]. It is possible that due to the absence of CDT,*Cjd *strains fail to induce the release of IL-8 and this may be responsible partly for the much higher incidence of *Cjd *in blood cultures compared to *Cjj*. Interestingly, deletions of portions of *cdtA *and *cdtB *have recently been reported for three CDT-negative *C. jejuni *strains that were also isolated from patients with bacteremia [40]. This further supports a relationship between the absence of *cdt *genes and bacteremia; however, it is not clear if the strains examined in that study were *Cjj *or *Cjd*. Additionally, CGH experiments comparing 11 [18], 18 [21], or 51 [22]*Cjj *strains reported complete conservation of the *cdt *locus across this subspecies, suggesting that production of CDT may be yet another marker that distinguishes *Cjj *and *Cjd*.

## Conclusion

Although *Cjd *strains have been distinguished from *Cjj *strains, due to phenotypic characteristics associated with *Cjd *strains, including the inability to reduce nitrate, high susceptibility to cephalothin and variable growth at 42°C [3], the genomic relationship between these subspecies has not been reported previously. Two types of genomic relationships between the two subspecies were possible: first, that the *Cjd *strains represented a phylogenetically- and phenotypically-distinct clade within *C. jejuni *and second, that the two subspecies were indistinguishable phylogenetically but possessed unique phenotypic characteristics. Initial studies that differentiated Campylobacters by amplified fragment length polymorphism (AFLP) profiling [29,30] suggested that the first possibility was the most likely. Consistent with these observations, using MLST and CGI analysis, we confirmed in this study that *Cjd *strains are phylogenetically distinct from *Cjj *strains. Indeed, all of the *Cjd *strains examined from our geographically diverse collection had the same *aspA63 *allele and possessed a common set of absent or highly variable genes. This set of genes included both genes in the intrastrain HV regions and 21 genes outside these regions. Moreover, we recently demonstrated that the loss of nitrate reductase activity in 27 *Cjd *strains was the result of an identical 2761 bp deletion in the nitrate reductase large subunit-encoding gene, *napA *[35]. Also, there is a high frequency of novel MLST alleles among the *Cjd *strains. Together, these analyses suggest that *Cjd *strains arose from a common ancestor. Considering *C. jejuni *is generally a non-clonal organism with a high frequency of gene exchange, it is possible that *Cjd *strains are ecologically isolated from *Cjj *strains, with MLST alleles arising mainly by mutation rather than recombination. However, it is also possible that *Cjd *strains share the same environment with *Cjj *strains but have a barrier to recombination. Characterization of additional *Cjd *strains might provide clues as to the potential evolutionary origin of *Cjd *and their possible ecological niche.

It is not clear how the common genotypic characteristics of *Cjd*, identified in this study, relate to the observed clinical characteristics such as bacteremia [3]. Nevertheless, the absence of the virulence related genes, *cdtA *and *cdtB *may play a role. It is also possible that there are unidentified virulence-related genes present in the *Cjd *strains that are absent from *Cjj *strains. The current CGI analysis measures the gene content of the *Cjd *strains relative to the NCTC 11168 and RM1221 genes present on the microarray; thus, *Cjd*-specific genes would not be detected by the CGI analysis. The current genomic sequencing project of *Cjd *strain 269.97 by J. Craig Venter Institute (JCVI) may identify such unique *Cjd *genes [41]. It is also possible that many of the differences between the two subspecies are a result of more subtle differences. These subtle differences could be bp changes that result in proteins with a different topology or substrate specificity or bp changes that affect gene regulation or protein expression) that would not be detected using an amplicon-based DNA microarray with CGI analysis but would be observed from the sequence data. Additional, comparative genomic analysis will be possible once the sequence is available.

## Methods

### Bacterial strains, growth conditions and chemicals

*Campylobacter *strains used in this study are listed in Table 1. All *Cjd *strains were cultured routinely at 37°C under microaerophilic conditions (5% O_2_, 10% CO_2_, and 85%N_2_) on Anaerobe basal agar (ABA; Oxoid, Basingstoke, UK) amended with 5% (v/v) laked horse blood (Hema Resource & Supply, Aurora, OR). All chemicals were purchased from Sigma-Aldrich (St. Louis, MO)or Fisher Scientific (Houston, TX). PCR enzymes and reagents were purchased from New England Biolabs (Beverly, MA) or Epicentre(Madison, WI). DNA sequencing chemicals and capillaries were purchased from Applied Biosystems (Foster City, CA). Sequencing and PCR oligonucleotides were purchased from MWG-Biotech (High Point, NC).

### Phenotypic characterization of *C. jejuni *subsp. *doylei *strains

To determine nitrate reduction in *C. jejuni*, nitrate disks and anaerobic nitrate reagents A and B (Remel, Lenexa, KS) were used. Zinc dust was obtained from BioMérieux (Durham, NC). *Cjj *strain NCTC 11168 and *Cjd *strain ATCC 49350 were used as controls for the procedure. Each *Cjd *strain was streaked onto ABA agar supplemented with 5% horse blood and grown for 48 h as described above. A nitrate disk was then placed on a thick zone of growth on the plate and incubated for an additional 24 h under the same conditions. The nitrate disk was removed subsequently and placed in a sterile tube. One drop of both reagents A and B were added to the disk. Reduction of nitrate is indicated by a color change (clear to red); if no color change was observed after 3 min, zinc dust was added. A color change prior to the addition of zinc is indicative of *Cjj *and a color change only after addition of zinc is indicative of *Cjd*.

### Multilocus sequence typing of *Cjd *isolates

The *Cjd *isolates in Table 1 were typed as described previously [6,42], using the *Cj*/*Cc *MLST primer sets. An in-house Perl program, MLSTparser was used to extract allele sequences as described previously [42]. All allelic sequences were queried against the *Campylobacter jejuni/coli *MLST database [43]. Alleles already present in the database were assigned those numbers; novel alleles and sequence types (STs) were submitted to the *Campylobacter jejuni/coli *MLST database and assigned new numbers. Each novel *Cjd *ST was compared to existing *Cjj *STs by concatenating the allelic sequences at all loci for that ST and performing either pairwise BLASTN comparisons against similarly concatenated *Cjj *ST sequences or performing CLUSTALX alignments with subsequent phylogenetic analysis. eBURST analysis was performed using the Imperial College, London eBURST v3 web site [44] and the *Campylobacter jejuni *dataset with a group definition of four.

### Fluorescent labeling of genomic DNA and microarray hybridization

*Cjd *genomic DNA was prepared using the Wizard Genomic DNA kit (Promega, Madison, WI) according to the manufacturer's protocols. Fluorescent labeling reactions of genomic DNA were performed as described previously [23]. Briefly, genomic DNA from the reference strains (*Cjj *strain NCTC 11168 and *Cjj *strain RM1221) and a test strain were labeled fluorescently with indodicarbocyanine (Cy5)-dUTP and indocarbocyanine (Cy3)-dUTP, respectively. Approximately 2 μg of DNA was mixed with 5 μl 10 × NEBlot labeling buffer, containing random sequence octamer oligonucleotides (New England Biolabs), and water to a final volume of 41 μl. This mixture was heated to 95°C for 5 min and then cooled for 5 min at 4°C. After this time, the remainder of the labeling reaction components were added: 5 μl of 10 × dNTP labeling mix (1.2 mM each dATP, dGTP, dCTP; 0.5 mM dTTP in 10 mM Tris pH 8.0; 1 mM EDTA), 3 μl of Cy3 dUTP or Cy5 dUTP (GE Biosciences, Piscataway, NJ) and 1 μl of Klenow fragment. The labeling reactions were incubated overnight at 37°C. Labeled DNA was purified from unincorporated label using Qiaquick PCR Cleanup kits (Qiagen). Labeling efficiencies were determined using a NanoDrop ND-1000 (NanoDrop, Wilmington, DE) and efficiently-labeled samples were dried by vacuum.

Microarray hybridization reactions were performed as described previously [23]. Fluorescently-labeled reference and test DNAs were combined in 45 μl Pronto! cDNA hybridization solution (Corning, Corning, NY) and heated to 95°C for 5 min. Fifteen μl of the hybridization mixture was applied to a microarray and sealed with a coverslip. The microarray slide was placed in a hybridization chamber (Corning) and incubated at 42°C for 18 h. Following hybridization, the slides were washed twice in 2 × SSC, 0.1% sodium dodecyl sulfate at 42°C for 10 min, twice in 1 × SSC at room temperature for 10 min, and finally twice in 0.2 × SSC at room temperature for 5 min. The microarray slides were dried by centrifugation at 300 × *g *for 10 min before scanning. At least two hybridization reactions were performed for each test strain.

### DNA microarray data acquisition and analysis

We recently described the construction and utilization of the multi-strain *C. jejuni *microarray in a comparative genomic hybridization (CGH) study [23]. Briefly, the multi-strain *C. jejuni* microarray contains 1530 PCR-amplified sequences from the annotated open reading frames (ORFs) of *C. jejuni *strain NCTC 11168 and 227 PCR-amplified sequences representing novel *C. jejuni *strain RM1221 ORFs spotted in duplicate onto Ultra-GAPS glass slides (Corning Inc., Corning, NY).

DNA microarrays were scanned using an Axon GenePix 4000B microarray laser scanner (Axon Instruments, Inc. Union City, CA). Features and the local background intensities were detected and quantified with GenePix 4.0 software (Axon Instruments, Inc.). Poor features were excluded from further analysis if they contained abnormalities or were within regions of high fluorescent background. The data were filtered so that spots with a reference signal lower than the background plus 2 standard deviations of the background were discarded. Signal intensities were corrected by subtracting the local background, and then the Cy5/Cy3 ratios were calculated. To compensate for unequal dye incorporation, data normalization was performed as described previously [23,45]. Dye-swapping was performed for selected strains and no effects due to differences in dye incorporation on downstream analysis were observed. The presence or absence of genes from *Cjj *strain NCTC 11168 and *Cjj *strain RM1221 in the other *Cjj *and *Cjd *strains was determined based on a comparison of normalized hybridization signal ratios of the test strain to the combined reference strains *Cjj *strain NCTC 11168 and *Cjj *strain RM1221 for the respective gene spots. The NCTC 11168 and RM1221 strain-specific spots hybridized to only half of the reference DNA (Cy5-labeled mixture of NCTC 11168 and RM1221 DNA), increasing the Cy3/Cy5 ratio 2 fold. Therefore, the ratios for these spots were divided by 2 before determining the status of the gene. The ratios for spots of each individual gene were then averaged. As previously described [23], we defined the status of a gene as present when the Cy3/Cy5 (test/reference) intensity ratio was > 0.6, as divergent/unknown when the Cy3/Cy5 intensity ratio was between 0.6 and 0.3, and absent when the Cy3/Cy5 intensity ratio was < 0.3. The presence, divergence and absence status for all genes was converted into trinary scores (present = 2; divergent/unknown = 1; absent = 0). The trinary gene scores for each replicate for all strains were analyzed further with GeneSpring microarray analysis software version 7.3 (Agilent Technologies, Redwood City, CA) and subjected to average-linkage hierarchical clustering with the standard correlation and bootstrapping.

## Authors' contributions

CTP, WGM and AJL designed the research project. WGM performed and analyzed the MLST. CTP and STH constructed the *C. jejuni *DNA microarray and performed the microarray experiments. CTP performed CGI analysis on the microarray data and drafted the manuscript. All authors approved and read the final manuscript.

## Supplementary Material

Additional file 1 Genomic indexing of *Cjd *strains. The data provided represent CGI data sets for the *Cjd *strains as trinary scores. Click here for file

## Figures and Tables

**Table 1:** *Campylobacter *strains used in this study

**Strain**	**Description**	**Location**	**Reference or source**
1. NCTC 11168	*Campylobacter jejuni *subsp. *jejuni*; Lior 4, Penner HS:2	UK	Human clinical isolate; [48].
2. RM1221	*Campylobacter jejuni *subsp. *jejuni*; Penner HS:53	USA	Chicken carcass; [34].
3. ATCC 49350	*Campylobacter jejuni *subsp. *doylei*;	Germany	Human clinical isolate.
4. ATCC 49351	*Campylobacter jejuni *subsp. *doylei*	Australia	Human clinical isolate.
5. CCUG 18266	*Campylobacter jejuni *subsp. *doylei*	Germany	Human clinical isolate.
6. RM2095	*Campylobacter jejuni *subsp. *doylei*	USA	Mabel Nicholson; human blood.
7. RM2096	*Campylobacter jejuni *subsp. *doylei*	USA	Mabel Nicholson; human clinical isolate.
8. RM3782	*Campylobacter jejuni *subsp. *doylei*	South Africa	Human clinical isolate.
9. SSI 5384	*Campylobacter jejuni *subsp. *doylei*	Denmark	Stephen On; human clinical isolate.
10. 269.97	*Campylobacter jejuni *subsp. *doylei*	South Africa	Human blood.

**Table 2:** Genomic regions variable between *C. jejuni *subsp. *jejuni *and *C. jejuni *subsp. *doylei *strains

		ATCC	CCUG	ATCC	RM	RM	SSI	RM	
NCTC 11168 gene(s)	Product(s)^a^	49350	18266	49351	2095	3782	5384	2096	269.97

Cj0005c	Putative molybdenum-containing oxidoreductase	**-**	**-**	**-**	**-**	**-**	**-**	**-**	**-**
Cj0031	Type IIS restriction/modification enzyme	**-**	**-**	**-**	**+**	**+**	**+**	**-**	**-**
Cj0046	Probable transport protein (pseudogene)	**-**	**-**	**+**	**-**	**-**	**-**	**-**	**-**
Cj0078c	Cytolethal distending toxin subunit CdtB	**-**	**-**	**-**	**-**	**-**	**-**	**-**	**+**
Cj0079c	Cytolethal distending toxin subunit CdtA	**-**	**-**	**-**	**-**	**-**	**-**	**-**	**-**
Cj0091	CH lipoprotein	**-**	**-**	**-**	**-**	**-**	**-**	**-**	**-**
Cj0201c – Cj0202c	CH protein (2)	**-**	**-**	**-**	**-**	**-**	**-**	**-**	**-**
Cj0203	Probable Mg^2+^/citrate complex transporter	**-**	**-**	**-**	**-**	**-**	**+**	**-**	**+**
Cj0224	N-acetyl-γ-glutamylphosphate reductase Arg C	**-**	**-**	**-**	**-**	**-**	**-**	**-**	**+**
Cj0226	Acetylglutamate kinase Arg B	**-**	**-**	**-**	**-**	**-**	**-**	**-**	**+**
Cj0339 – Cj0340	MFS transporter, nucleoside hydrolase	**-**	**-**	**-**	**-**	**-**	**-**	**-**	**-**
Cj0548	Flagellar hook-associated protein FliD	**-**	**-**	**-**	**-**	**+**	**-**	**+**	**+**
Cj0555	CH membrane protein	**-**	**-**	**-**	**+**	**-**	**-**	**-**	**-**
Cj0556	CH protein (putative amidohydrolase)	**-**	**-**	**-**	**+**	**+**	**-**	**-**	**+**
Cj0558c	γ-glutamylphosphate reductase ProA	**-**	**-**	**-**	**-**	**-**	**-**	**-**	**-**
Cj0636	Sun/nucleolar protein family protein	**-**	**-**	**+/-**	**-**	**-**	**-**	**-**	**-**
Cj0671	C4-dicarboxylate transporter DcuB	**-**	**-**	**-**	**-**	**-**	**-**	**-**	**-**
Cj0685c	CH protein (possible sugar transferase)	**-**	**-**	**-**	**+**	**+**	**+**	**+**	**-**
Cj0778	Major antigenic peptide PEB2	**-**	**-**	**-**	**-**	**-**	**-**	**-**	**+**
Cj0780	Nitrate reductase, large subunit NapA	**-**	**-**	**-**	**-**	**-**	**-**	**-**	**-**
Cj0783	Nitrate reductase, small subunit NapB	**+**	**+**	**+**	**-**	**-**	**-**	**+**	**-**
Cj0859c – Cj0860	CH protein, CH membrane protein	**+**	**+**	**+**	**-**	**-**	**-**	**-**	**+**
Cj0878	CH protein	**+**	**+**	**+**	**+**	**-**	**-**	**-**	**-**
Cj0937 – Cj0938c	CH membrane protein, acyltransferase Aas	**-**	**-**	**-**	**-**	**-**	**-**	**-**	**-**
Cj0989	CH protein	**-**	**-**	**-**	**-**	**-**	**-**	**-**	**-**
Cj0990c	CH protein	**-**	**+/-**	**-**	**-**	**+**	**+**	**-**	**+**
Cj0999c – Cj1000	CH membrane protein, transcriptional regulator	**-**	**-**	**-**	**-**	**-**	**-**	**-**	**-**
Cj1040c – Cj1042c	transporter, CH protein, transcriptional regulator	**-**	**-**	**-**	**-**	**-**	**-**	**-**	**-**
Cj1165c	CH membrane protein	**-**	**-**	**-**	**+**	**+**	**-**	**+**	**-**
Cj1166c	CH membrane protein	**-**	**-**	**-**	**-**	**+**	**-**	**+**	**-**
Cj1168c	CH membrane protein	**-**	**-**	**-**	**+**	**-**	**-**	**+**	**-**
Cj1353	Iron ABC transporter protein CeuC	**-**	**-**	**-**	**-**	**-**	**-**	**-**	**-**
Cj1354	Iron ABC transporter protein CeuD	**-**	**-**	**-**	**-**	**-**	**-**	**+**	**-**
Cj1365c	Serine protease	**-**	**-**	**-**	**-**	**-**	**-**	**-**	**-**
Cj1456c	CH protein	**-**	**-**	**-**	**-**	**-**	**-**	**+**	**+**
Cj1546	Transcriptional regulator	**+**	**-**	**-**	**-**	**+**	**+**	**+**	**-**
Cj1564	MCP	**-**	**-**	**-**	**-**	**-**	**-**	**-**	**-**
Cj1602	CH protein	**+**	**+**	**+**	**-**	**-**	**-**	**+**	**-**
Cj1625c	L-serine transporter SdaC	**-**	**-**	**-**	**-**	**-**	**-**	**-**	**-**

^a^. CH: conserved hypothetical, MCP: methyl-accepting chemotaxis protein.
